# Laser Spectroscopy of Aromatic Molecules with Optical Cycling Centers: Strontium(I) Phenoxides

**DOI:** 10.1021/acs.jpclett.2c03040

**Published:** 2022-11-22

**Authors:** Guanming Lao, Guo-Zhu Zhu, Claire E. Dickerson, Benjamin L. Augenbraun, Anastassia N. Alexandrova, Justin R. Caram, Eric R. Hudson, Wesley C. Campbell

**Affiliations:** † Department of Physics & Astronomy, University of California Los Angeles, Los Angeles, California90095, United States; ‡ Department of Chemistry & Biochemistry, University of California Los Angeles, Los Angeles, California90095, United States; ¶ Department of Physics, Harvard University, Cambridge, Massachusetts02138, United States; § Harvard-MIT Center for Ultracold Atoms, Cambridge, Massachusetts02138, United States; ∥ Center for Quantum Science and Engineering, University of California, Los Angeles, California90095, United States; ⊥ Challenge Institute for Quantum Computation, University of California, Los Angeles, California90095, United States

## Abstract

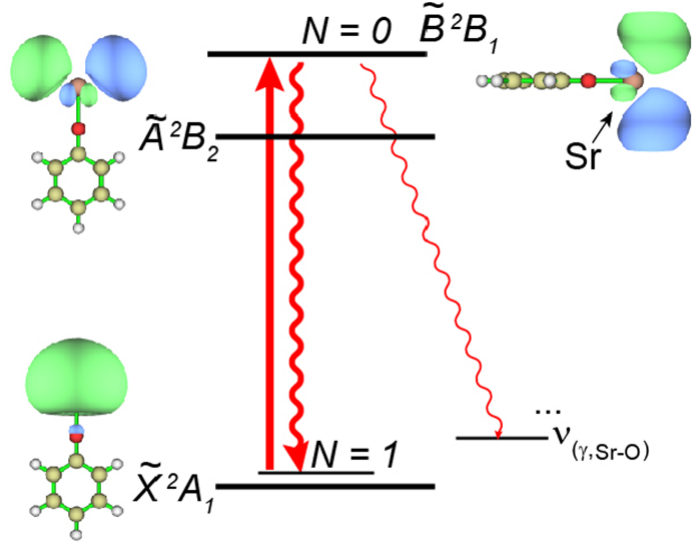

We report the production and spectroscopic characterization of strontium(I) phenoxide (SrOC_6_H_5_ or SrOPh) and variants featuring electron-withdrawing groups designed to suppress vibrational excitation during spontaneous emission from the electronically excited state. Optical cycling closure of these species, which is the decoupling of the vibrational state changes from spontaneous optical decay, is found by dispersed laser-induced fluorescence spectroscopy to be high, in accordance with theoretical predictions. A high-resolution, rotationally resolved laser excitation spectrum is recorded for SrOPh, allowing the estimation of spectroscopic constants and identification of candidate optical cycling transitions for future work. The results confirm the promise of strontium phenoxides for laser cooling and quantum state detection at the single-molecule level.

Optical cycling transitions in atoms allow laser cooling of the center-of-mass motion, laser state preparation, and laser-induced fluorescence (LIF) state detection—open-channel operations at the heart of many promising applications of quantum technology, including quantum computation,^1,2^ atomic clocks,^3,4^ and quantum simulation.^5,6^ Optical cycling and cooling schemes have also been demonstrated in diatomic^7,8^ and even some small polyatomic molecules,^9,10^ including SrF,^11^ YO,^12^ CaF,^13,14^ YbF,^15^ BaF,^16,17^ MgF,^18^ AlF,^19^ SrOH,^20^ CaOH,^21,22^ YbOH,^23^ and CaOCH_3_.^24^ Because they possess rich internal structures and complex interactions, molecules provide new opportunities in studies of dark matter detection,^25,26^ measurement of electron’s electric-dipole moment,^27−29^ parity violation tests,^30,31^ and changes to fundamental constants.^32,33^ The somewhat unexpected atom-like transitions supporting optical cycling and cooling in these small molecules have inspired searches for similar transitions in complex polyatomic molecules with an M–O–R structure,^9,10,34−40^ where M is an alkaline-earth metal atom ionically bonded to oxygen (O) forming an optical cycling center (OCC) and R is a molecular ligand.^36−40^ In these molecules, the remaining metal-centered radical electron forms the highest-occupied and the lowest-unoccupied molecular orbitals, HOMO and LUMO. For molecules with R having strong electron-withdrawing capability, the HOMO and LUMO are localized on M, which typically indicates that the OCC is highly decoupled from the vibrational degrees of freedom. As a result, the diagonal vibrational branching ratio (VBR, which is to say the probability that spontaneous decay occurs on the 0–0 transition) is high, indicating that the spontaneous emission happens without a vibrational state change. This allows such molecules to repeatedly scatter photons before being pumped to the vibrational dark states, furnishing mechanical control and state detection of single molecules via laser illumination.

Since optical cycling in this motif is predicted to be enhanced by the electron-withdrawing strength of the ligand, the diagonal VBR of M–O–R molecules could be tuned by functionalizing the ligand to promote this effect.^34,39^ For example, according to a recent measurement of the VBRs,^41^ laser cooling of CaOPh-3,4,5-F_3_ (Ph, phenyl group) appears feasible from the perspective that each molecule could scatter ≈1000 photons with six to eight lasers. Compared to CaOPh, the three substitutions of H → F in the 3, 4, and 5 positions on the ring enhance the electron-withdrawing strength of the ligand, rendering the Ca atom more ionic and thus suppressing spontaneous decays to excited vibrational states of the electronic ground state.

As molecules of M–O–R type, the strontinum variants, SrOPh-X, were also predicted to have high and tunable diagonal VBRs.^39^ Compared to CaOPh-X, although the diagonal VBRs were predicted to be lower, the predicted difference is of the same order as the variation in measured VBRs of various calcium species,^41^ suggesting that some of the strontium species may show better cycle closure if the variation is due to M-specific features. Further, Sr-containing molecules allow exploration of the role of strong spin–orbit coupling^42^ and nuclear spin structures.^43^ For the strontium variants, the excitation and repumping wavelengths can be directly produced by diode lasers.

Here, we report the production and spectroscopic characterization of strontium(I) phenoxide (SrOPh) and its derivatives, SrOPh-X (X = 3-CH_3_, 3-F, 3-CF_3_, and 3,4,5-F_3_, see Scheme 1). Gas-phase molecules are produced by the reaction of Sr atoms generated by the ablation of Sr metal with the corresponding organic precursor vapor and cooled via collisions with the neon buffer gas in a cryogenic cell at a temperature of ≈23 K. The first two electronically excited states, which have been theoretically proposed for optical cycling and laser cooling, are identified and the respective vibrational decays are observed using the dispersed laser-induced fluorescence (DLIF) spectroscopy. Details of the experimental and theoretical methods can be found in the Supporting Information.^44^ The diagonal vibrational branching ratios are estimated to be 0.82–0.96, which indicates promise for laser cooling with a handful of vibrational repump lasers. To further characterize candidate optical cycling transitions, we have measured the rotationally resolved excitation spectrum for the  transition of SrOPh and obtained the molecular constants by fitting using PGOPHER.^45^

**Scheme 1:**
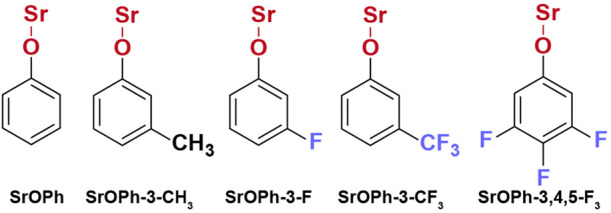
Molecular Structures of Strontium(I) Phenoxide and Its Derivatives Studied in This Work

In the calcium and strontium phenoxides, transitions to the two lowest electronic states ( and , Figure 1a) have been proposed for laser cooling, since almost all photon scatters go back to the vibrationless ground state .^39,41,46^Figure 1b shows the measured transition energies of all molecules show a linear correlation with the acid dissociation constants, p*K*_a_, of the precursor phenol. This linear trend has recently also been observed for CaOPh-X molecules.^41,46^ A lower p*K*_a_ implies higher electron-withdrawing capability of the R–O^–^ ligand, which pulls the single electron away from the Sr atom, making it more ionic and increasing the HOMO–LUMO gap.^39^ Also shown are excitation energies calculated by time-dependent density functional theory (TD-DFT)^44^ which give a similar trend but systematically undershoot the excitation energies likely due to self-interaction error and approximate treatment of electronic correlation.^47^ The calculated energy gap of  (36–68 cm^–1^) is much smaller than the measured gap (300–324 cm^–1^), similar to what was observed in CaOPh-X species but with a wider difference between the theory and measurement.^41^ The theory–experiment discrepancies of the  energy gap are likely due to the lack of spin–orbit coupling (SOC) in calculations^48^ and the wider difference in SrOPh-X is due to the stronger SOC effects in Sr.

**Figure 1:**
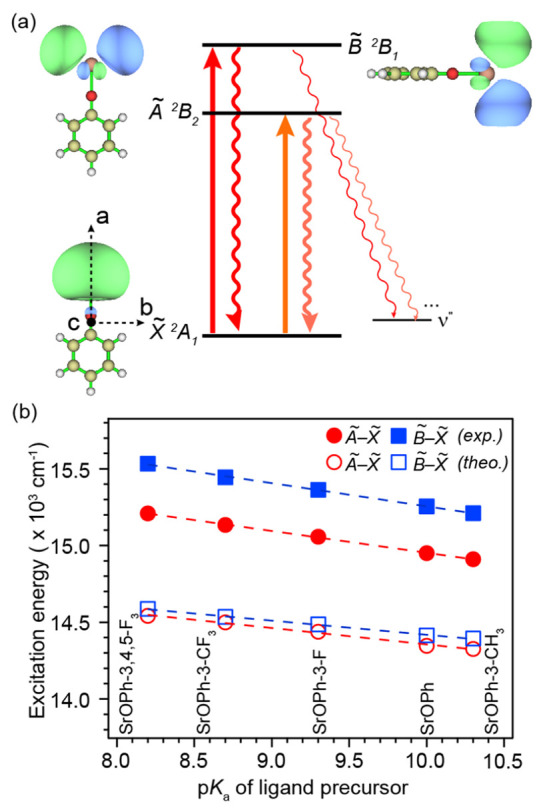
(a) Schematic energy levels of the transitions proposed for laser cooling. The molecular orbital and the respective symmetry of each state are illustrated for SrOPh with a *C*_2*v*_ point group. For molecules with *C*_*s*_ symmetry, the symmetries would be A′ , A″, and A′(). The principle inertial axes are also given. (b) Excitation energy versus p*K*_a_ for  and  transitions for all studied species in an increasing order of ligand p*K*_a_.^41^ The linear fits of the experimental values yield  (16380 – 142.8 × p*K*_a_) cm^–1^ and = (16777 – 152.3 × p*K*_a_) cm^–1^.

To measure the VBRs from the two electronic states, we performed DLIF spectroscopy of all molecules. Electronic excitation is provided by a pulsed dye laser (PDL) tuned to the 0–0 line, and the spectrometer grating was scanned in time (over repeated excitation) to select the wavelength of LIF photons sent to a photomultiplier tube (PMT).^44^Figure 2 shows the measured DLIF spectra of SrOPh while those of other species are presented in Figure S1. Figure 2a shows the spectrum of Ã ^2^B_2_ → X̃ ^2^A_1_ of SrOPh (Figure 1a) at an excitation of 669.06 nm. The strongest peak at the origin, labeled as , is due to the diagonal decay from  to . The strong peak at −440 cm^–1^ is from excited atomic Sr created during laser ablation.^49^ The peak at −238 cm^–1^ is assigned to the strongest off-diagonal stretching mode ν_3_ (theory 241 cm^–1^) and the weak peak at −54 cm^–1^ is assigned to the low-frequency bending mode ν_2_ (theory 56 cm^–1^). The other two weak peaks at −100 cm^–1^ and −297 cm^–1^, which do not match the calculated frequencies of any fundamental vibrational modes, are assigned to the overtone of the bending mode ^A^2_2_^0^ and a combinational mode of , respectively.

**Figure 2:**
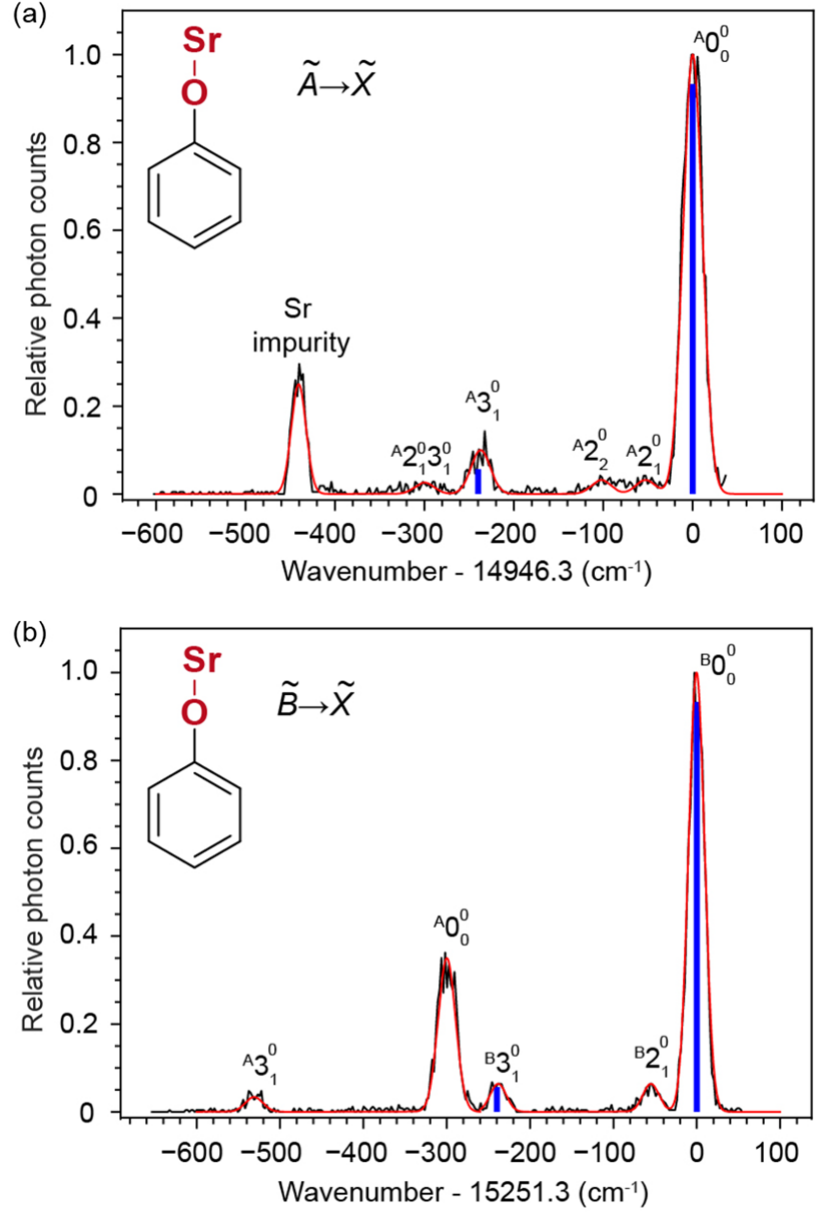
(a) and (b) Dispersed spectra of  and , respectively, of SrOPh excited by pulsed dye laser and measured by a spectrometer coupled with PMT. The experimental curves (black) are fitted with the Gaussian functions (red). The positions of the blue, vertical lines illustrate the theoretical frequencies while the intensities show the vibrational branching ratios of different vibrational modes of SrOPh. The Sr impurity peak in (a) is from the Sr emission of  at 689 nm.^49^ The assignments of all resolved vibrational peaks are indicated.

Figure 2b shows the spectrum of B̃ ^2^B_1_ → X̃ ^2^A_1_ of SrOPh (Figure 1a) at 655.68 nm. Aside from the strongest diagonal peak , four peaks are observed. The strong peak with a shift of −300 cm^–1^ is due to a diagonal decay  from the  state. The origin of the appearance of  when exciting the  is unknown but could be due to the collisional relaxation from  to  followed by fluorescence decay to the ground state .^41,46,50^ The identification of this feature as originating from the  state is further confirmed by the observation of the decay to the stretching mode ν_3_ at −534 cm^–1^ from . The other two weak peaks, −238 cm^–1^ and −55 cm^–1^, are due to the vibrational decay to the stretching mode ν_3_ and bending mode ν_2_, respectively. The full width at half-maximum of all peaks is ≈22 cm^–1^ mainly due to the spectrometer resolution of approximately 20 cm^–1^. Another measurement was performed using a narrow-band continuous-wave (cw) laser to excite the  of SrOPh and an electron-multiplying charge-coupled device (EMCCD) camera to capture the fluorescence photons dispersed by the spectrometer. This technique obtained a better spectral resolution (≈5 cm^–1^), allowing the resolution of the combinational vibrational mode of  at −300 cm^−1^ (Figure S2), which is overlapped with the diagonal decay  from the  state and not observed in Figure 2b. The experimental and theoretical vibrational frequencies of all resolved fundamental modes are summarized in Table S1.

The relative heights of the peaks  and  in Figure 2 and Figures S1 and S2 imply that both transitions are very diagonal with few vibration-changing decays. To extract the VBRs, all peaks are fitted with Gaussian functions, as shown by the red traces in Figure 2, and the peak areas are extracted from the fits to obtain VBRs. A strict definition of VBR requires measurements of all vibrational decays. Due to finite measurement sensitivity  and detection range (<600 cm^–1^), while we predict that our measurement is sensitive to the dominant leakage channels, the possibility of undetected decays contributes a systematic uncertainty on the measured VBRs.

For the vibrational decays that were identified for each molecule, and the ratios of line intensities to the total intensities of all *observed* peaks are presented in Figure 3a. In both electronic transitions, the relative ratios of observed peaks show good agreement with the calculated VBRs.^44^ The vibrational decays to the strongest off-diagonal Sr–O stretching mode (ν_3_, ν_4_, ν_5_, or ν_6_) and the low-frequency bending mode (ν_1_ or ν_2_) have been observed for all molecules. The theoretical VBRs of the low-frequency bending modes are underestimated, possibly due to the vibronic coupling and anharmonicity effect not considered in the calculation.^39,41^ SrOPh also shows unpredicted decays to the overtone of mode ν_2_ and a combinational mode ν_2_ν_3_ where the intensities could be from the vibronic coupling. The intensity ratios of all observed decays are summarized in Table S2. Figure 3b plots the estimated VBRs of the diagonal peak 0_0_^0^ of each transition as a function of ligand p*K*_a_. The scaled 0_0_^0^ VBRs are obtained by adding the estimated contribution of the unobserved peaks predicted by the theory to the normalized intensity ratios of the observed 0_0_^0^ intensities.^44^ Both SrOPh-3-F and SrOPh-3,4,5-F_3_ molecules show VBRs >95% for the  transition and >90% for the  transition, while SrOPh has the lowest VBR of 82.2% for  transition. Due to the predominantly localized excitations and previous benchmarking results,^39−41,44,46^ we find TD-DFT is sufficient to predict VBR trends in SrOPh optical cycling species. However, we find our theoretical calculations still lack important dynamic correlation and spin–orbit coupling which will affect important branching pathways. For high-level predictions beyond simple trends, we suggest choosing methods which can improve upon dynamic correlation systematically, such as coupled-cluster,^34−36^ and incorporating the Breit–Pauli operator to compute spin–orbit coupling effects.^51^

**Figure 3:**
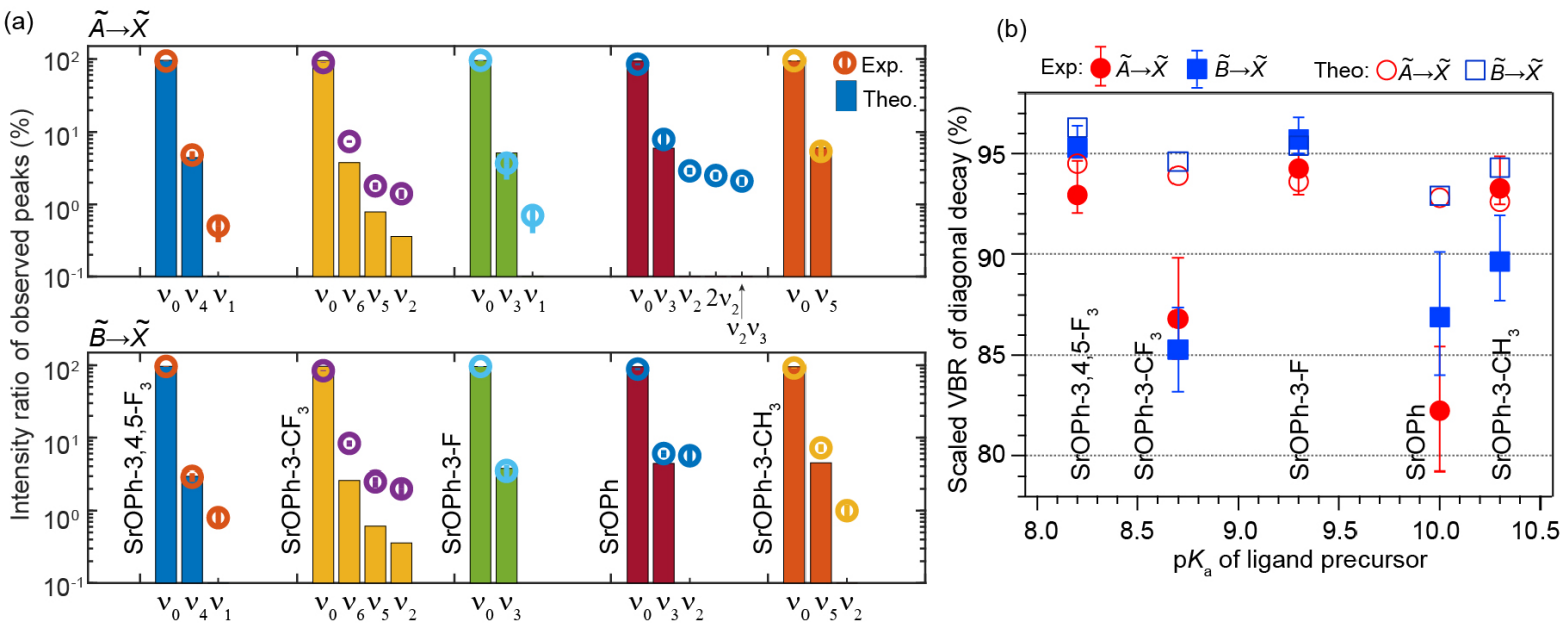
(a) Intensity ratio of observed decays for  and  transitions. Error bars are statistical errors from Gaussian fits. The vibrational label ν_*i*_ indicates the final vibrational modes of the  state. ν_0_ implys the decay that does not change the vibrational state. (b) Scaled 0_0_^0^ VBRs as a function of p*K*_a_ of all species. The scaling adds the contributions of those unobserved vibrational decays predicted by the theory to the observed intensity ratios of 0_0_^0^ in (a). Error bars include the statistical errors from Gaussian fits and the systematic errors from the unobserved peaks.^44^

The VBRs for SrOPh-3-CF_3_ shows the largest discrepancy between the calculation and the measurement, potentially due to the larger vibronic mixing between the  and  caused by the low symmetry and large electron inductive effect from the CF_3_ group.^41^ The error bars include both the statistical uncertainties from the Gaussian fit and the systematic uncertainty estimate from the unobserved peaks. Three additional systematic errors, including signal drift during measurement, the wavelength response of the spectrometer, and the diagonal excitation from the vibrationally excited states, are estimated to be a few percent in total.^44^

To further investigate the potential of these species for optical cycling, a high-resolution excitation spectrum (obtained by collecting LIF as a continuous-wave (cw) excitation laser is scanned) of SrOPh for the  transition is recorded at a step size of 25–50 MHz in a cryogenic buffer-gas beam (CBGB)^44,52^ and fitted with PGOPHER,^45^ as presented in Figure 4. Since SrOPh is an asymmetric-top molecule, the rotational states are labeled as , where *N* is the rotational angular momentum and *a* and *c* label the inertial axes lying along the Sr–O bond and perpendicular to the molecular plane (Figure 1a), respectively, *K*_*a*_ and *K*_*c*_ are the projection of *N* onto the two axes in the prolate and oblate limits, respectively. Figure 4a shows the expansion of the two congested bands at 15238.5 cm^–1^, while Figure 4 panels b and c show two well-resolved rotational bands. A full rotational analysis is difficult due to the high density of rotational lines in the middle of the spectrum (Figure 4a), but the individually resolved lines (Figure 4b,c) make it possible to fit the spectrum to extract some spectroscopic constants.

**Figure 4:**
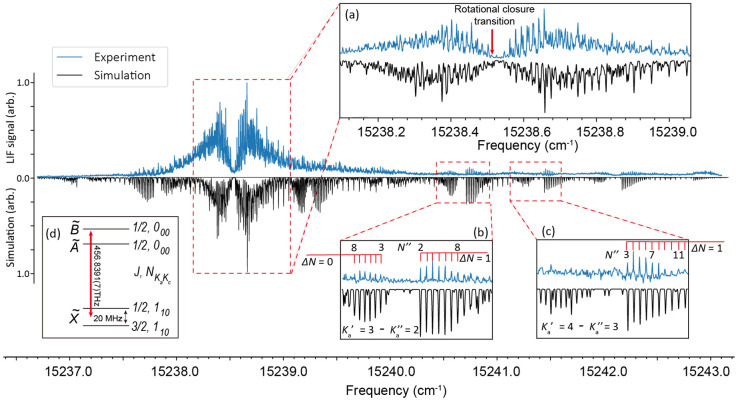
High-resolution rotationally resolved excitation spectrum of the  transition of SrOPh. The upper trace (blue) shows the experimental spectrum and the lower trace (black) is the simulated spectrum with a Gaussian line width of 70 MHz and a rotational temperature *T*_sim_ = 2.5 K. Insets a, b, and c are expansions of some local features. (a) displays detailed spectrum near 0–0 transition, while (b) and (c) show the *K*_*a*_^′^ = 3 ← *K*_*a*_^″^ = 2 and *K*_*a*_^′^ = 4 ← *K*_*a*_^″^ = 3 rotational bandheads, respectively. (d) shows the inferred position of the candidate rotational cycling transition between the spin-rotation manifold of the *N*″ = 1 state and *N*′ = 0 state.

Using a custom program to fit the spectral contour and PGOPHER(45) to refine and iterate the line assignments,^44^ we have assigned nearly 400 rotational transitions and obtained the final fitted spectrum given as the black traces in Figure 4. The fitting is in agreement with the experimental measurement for the middle broad bands and the *K*_*a*_^′^ = 3 ← *K*_*a*_^″^ = 2 and *K*_*a*_^′^ = 4 ← *K*_*a*_^″^ = 3 bands, as expanded in Figure 4a–c. The best fit molecular constants, including the transition energy, rotational constants, spin-rotation constants and centrifugal distortion corrections, are reported in Table 1. The measured rotational constants are in good agreement with the calculated values. The spin-rotation constant ϵ_*aa*_ in the ground state is too small to be determined from the spectrum, and ϵ_*aa*_ in the  state is large because of the coupling to the  state. The larger value of spin-rotation constant than the rotational constants in  implies a strong SOC effect apart from the direct coupling between the spin and molecular rotation. Based on the second order perturbation theory^53,54^ and the measured constants, the SOC constant in SrOPh is estimated to be ≈272 cm^–1^, which is close to that of SrOH (A^2^Π, ≈265 cm^–1^).^55^ The large SOC also dominates the energy separation of , elucidating the discrepancy between the calculation and the measurement in Figure 1b.^44,48^

**Table 1:** Molecular Constants of SrOPh Obtained by Fitting the Rotationally-Resolved Excitation Spectrum in Figure 4 with PGOPHER (All Quantities in cm^–1^)^a^

	B̃ ^2^B_1_		X̃ ^2^A_1_	
constant	exp	cal	exp	cal
*T*_0_	15238.7155(23)			
*A*	0.1923(6)	0.1915	0.1934(11)	0.1916
^1^/_2_(*B* + *C*)	0.01520(36)	0.01522	0.01508(36)	0.01513
(*B* – *C*) × 10^3^	1.28(20)	1.21	1.13(12)	1.19
ϵ_*aa*_	–0.6894(6)			
ϵ_*bb*_ × 10^3^	34(10)		1.3(1.7)	
ϵ_*cc*_ × 10^3^	16(7)		–1.3(1.8)	
*D*_*N*_ × 10^8^	–14(8)		–14(8)	
*D*_*NK*_ × 10^7^	–5(11)		–28(23)	
*D*_*K*_ × 10^4^	1.3(5)		5.2(1.1)	
*H*_*K*_ × 10^6^	3.0(1.4)		21(4)	

a *T*_0_: electronic transition energy. *A*, *B*, *C*: molecular rotational constants. ϵ_*aa*_, ϵ_*bb*_, ϵ_*cc*_: spin-rotation coupling constants. *D*_*N*_, *D*_*NK*_, *D*_*K*_: centrifugal distortion constants. *H*_*K*_: sextic centrifugal distortion correction.

While involving more parameters has been able to enhance the accuracy of fitting, many parameters in such scenarios tended to fit to values consistent with zero, and we therefore omit those in our analysis. The large error bars of some of the centrifugal distortion constants are mainly due to the uncertainty of the line assignment near the 0–0 transition. The rotational temperature from the fit is 2.5 K.^44^ The colder temperature is due to the free expansion of neon buffer gas from the cryogenic cell (≈23 K) to form a beam with SrOPh entrained.^52^ As the SrOPh  transition dipole moment lies along the principle axis *c* (Figure 1b), the rotationally closed photon cycling transition is the *c*-type transition ,^38^ which is estimated to be at 456.8391(7) THz based on the fitting results and shown in Figure 4a,d.

In summary, we have produced strontium(I) phenoxide (SrOPh) and derivatives featuring electron-withdrawing groups in a cryogenic cell. Two proposed laser cooling transitions ( and ) of each molecule have been identified and the transition energies show linear trends as the ligand p*K*_a_, which can be used to look for transitions of new molecules containing Sr. The overall vibrational branching ratios considering contributions of unobserved vibrational decays are estimated to be 82.2% to 95.8%. Among them, SrOPh-3-F and SrOPh-3,4,5-F_3_ molecules show diagonal VBRs >95%, potentially enabling laser cooling with fewer than ten vibrational repumping lasers. The rotationally resolved spectrum for the  transition of SrOPh is presented and molecular constants are obtained. The spin–orbit interaction that couples the  and  states is estimated to be 275 cm^–1^, which has a strong effect on the energy splitting of . The rotational closure transition for optical cycling is estimated to be centered near 456.8391(7) THz. This work paves the way for optical cycling of SrOPh and other large molecules using diode lasers.

## Supporting Information Available

Experimental and theoretical methods, discussion of DLIF spectra of other molecules, error analysis of VBRs, fitting of high-resolution excitation spectrum, spin–orbit coupling effect in SrOPh, Tables S1–S5 of frequencies, intensity ratios, systematic error budget, intensity ratios and scaled VBRs, and excitation energies and FC factors, and Figures S1–S4 of fluorescence spectra and decay traces and of line assignments (PDF)
Transparent Peer Review report available (PDF)
