# Supplementary material for: Laser Spectroscopy of Aromatic Molecules with Optical Cycling Centers: Strontium(I) Phenoxides
Source: J Phys Chem Lett. 2022 Nov 22;13(47):11029–35. doi: 10.1021/acs.jpclett.2c03040 (PMC9720742; doi:10.1021/acs.jpclett.2c03040)
Supplement: Supplementary file 1 — jz2c03040_si_001.pdf [file jz2c03040_si_001.pdf]

Supporting Information

for

Laser Spectroscopy of Aromatic Molecules with Optical Cycling Centers: Strontium (I) Phenoxides

Guanming Lao,[†] Guo-Zhu Zhu,[*,†] Claire E. Dickerson,[‡] Benjamin L. Augenbraun,[¶,§] Anastassia N. Alexandrova,[‡,‖] Justin R. Caram,[‡,‖] Eric R. Hudson,[†,‖,⊥] and Wesley C. Campbell[†,‖,⊥]

†*Department of Physics & Astronomy, University of California Los Angeles, Los Angeles, CA 90095, USA*

‡*Department of Chemistry & Biochemistry, University of California Los Angeles, Los Angeles, CA 90095, USA*

¶*Department of Physics, Harvard University, Cambridge, MA 02138, USA*

§*Harvard-MIT Center for Ultracold Atoms, Cambridge, MA 02138, USA*

‖*Center for Quantum Science and Engineering, University of California, Los Angeles, California 90095, USA*

⊥*Challenge Institute for Quantum Computation, University of California, Los Angeles, California 90095, USA*

E-mail: guozhu.zhu@physics.ucla.edu

Experimental methods

Molecule production. All SrOPh-X molecules were produced by the reaction of Sr atoms generated by laser ablation of a metallic Sr metal chunk using the Minilite pulsed Nd:YAG laser at 1064 nm (pulse energy $\approx$ 6 mJ, repetition rate 10 Hz) with different ligand precursors in a cryogenic buffer-gas cell operated at $\approx$ 23 K. Five ligands – phenol, *m*-cresol, 3-fluorophenol, 3-(trifluoromethyl)phenol and 3,4,5-trifluorophenol – purchased from Sigma Aldrich were individually heated in a reservoir to supply the respective vapors, which were guided via a heated gas line into the cryogenic cell with a density $\approx 10^{13}$ cm^{-3}. The reaction products were then cooled by colliding with a neon buffer gas of density $\approx 10^{15-16}$ cm^{-3}.

DLIF measurement in the cryogenic cell. The resulting SrOPh-X molecules have two low-lying electronic states proposed for laser cooling. To look for those states, a tunable, pulsed dye laser (10 Hz, LiopStar-E dye laser, linewidth 0.04 cm^{-1} at 620 nm) were used to excite molecules in the cryogenic cell and the laser wavelength was scanned. When the laser wavelength hit electronic resonance, the molecule was excited and followed by the emission of molecular fluorescence. The fluorescence was then collected via an imaging system into a model 2035 McPherson monochromator equipped with a 1200 lines/mm grating and detected by a PMT. The dispersed measurement were done by parking the laser wavelength at the electronic resonance and continuously scanning the grating of the spectrometer at an increment of 0.10 nm while monitoring the fluorescence photons. The entrance and exit slit widths were set at $\approx$ 0.5 mm and 0.2 mm, respectively, resulting in a spectrometer resolution of $\approx$ 20 cm^{-1}.

A different method was also used to measure the DLIF spectrum for SrOPh $\widetilde{B} \rightarrow \widetilde{X}$ transition (Figure S2). In this method, the SrOPh molecules were excited by a cw laser illuminated from a home-build external-cavity diode laser (ECDL). The fluorescence was dispersed by the monochromator at a fixed grating position and detected by an EMCCD camera. The entrance slit of the spectrometer was set to 0.03 mm to achieve a resolution of $\approx$ 3 cm^{-1}. In this measurement, all fluorescence photons were collected simultaneously by

the EMCCD, which avoided the systematic errors due to the ablation or PDL energy drift in scanning the grating in the first method.

High resolution excitation spectroscopy of SrOPh $\widetilde{B}-\widetilde{X}$. To look for the rotational closure transitions, We performed the high-resolution excitation spectroscopy of SrOPh in a molecular beam. SrOPh molecules formed in the cryogenic cell were extracted out via a 9 mm cell aperture and entrained into a neon buffer gas beam. The excitation zone is $\approx$ 23 cm downstream the cell aperture. A cw laser from an ECDL was scanned with an increment of $25-50$ MHz near the $\widetilde{B}(v'=0)-\widetilde{X}(v''=0)$ transition of SrOPh determined by the low-resolution PDL measurement. The fluorescence were collected by a PMT at the perpendicular direction. Due to the cooling effect in the expansion, SrOPh molecules in the beam are colder than those thermalized in the cell at a temperature $\approx$ 23 K.

Theoretical methods

Molecular geometries, excitation energies, and Franck-Condon factor (FCFs) calculations were performed in Gaussian16.[1] Density functional theory (DFT) with 6 roots in the Davidson algorithm search space was used for the ground states while time-dependent DFT was used for excited states. A superfine grid and very tight convergence parameters were used to optimize geometries with the PBE0-D3 functional (with dispersion corrections) and the def2-TZVPPD basis set.[2–5] An effective core potential (ECP) was used for the Sr atom within the def2-TZVPPD basis set. Molecular orbitals were generated with an isosurface of 0.03 in the Multiwfn program.[6] The FCFs were calculated between the ground and first few low-lying excited electronic states, within the Condon and Born-Oppenheimer approximations. The FCFs were computed within the harmonic approximation and included Duschinsky rotations, which appeared to be sufficient for an overall trend in FCF. However, the anharmonicity and vibronic coupling effects do play a key role in the the FCFs of the low-frequency bending modes,[7,8] which were underestimated here in comparison to the experimental measurements. As seen in Table S5, the long-range correted functional predicts more accurate FCFs and

excitation energies, however, PBE0-D3 is sufficient in predicting FCF trends, as both predict the same relative changes in excitation energy ($\approx$ 0.03 eV and FCF increase ($\approx$ 0.020 for wB97XD and $\approx$ 0.016 for PBE0-D3) from SrOPh and SrOPh-3,4,5-F_3. The calculated FCFs can be converted to the VBRs using the formula:[8,9]

$$\begin{aligned} b_{iv',fv''} &= \frac{A_{iv',fv''}}{\sum_{fv''} A_{iv',fv''}} \\ &= \frac{|\mu_{iv',fv''}|^2 \ \times \ (\nu_{iv',fv''})^3}{\sum_{fv''} |\mu_{iv',fv''}|^2 \ \times \ (\nu_{iv',fv''})^3} \\ &\approx \frac{\text{FCF}_{iv',fv''} \ \times \ \nu^3_{iv',fv''}}{\sum_{fv''} \text{FCF}_{iv',fv''} \ \times \ \nu^3_{iv',fv''}} \end{aligned} \tag{1}$$

where i and f imply the initial and final states, respectively. $b_{iv',fv''}$ is the branching ratio, $A_{iv',fv''}$ is the Einstein coefficient for spontaneous emission, $\mu_{iv',fv''}$ is the transition dipole moment and $\nu_{iv',fv''}$ is the transition frequency.

DLIF spectra of other molecules

Figure S1 presents the DLIF spectra of other four molecules, which were recorded by monitoring the fluorescence signal from the respective excited states when scanning the grating wavelength at a step size of 0.1 nm. All peaks were fitted with Gaussian functions, as shown by the red traces overlapped with the experimental black traces. Comparing to the theoretical vibrational frequencies and the respective VBRs (blue lines), those peaks can be readily assigned. Take SrOPh-3-CH_3 for an example, two peaks show up in the $\widetilde{A} \rightarrow \widetilde{X}$ decay in Figure S1a. The origin peak labeled as ${}^{A}0^0_0$ represents the decay of $\widetilde{A}(v'=0) \rightarrow \widetilde{X}(v''=0)$, while a weak peak at a frequency shift of -226 cm^{-1} matches well with the theoretical vibrational frequency of the Sr-O stretching mode ν_5 (theo. 230 cm^{-1}), which is also the most off-diagonal vibrational mode. In Figure S1b of $\widetilde{B} \rightarrow \widetilde{X}$ decay, except for the diagonal peak (${}^{B}0^0_0$) at the origin and the stretching-mode peak (${}^{B}5^0_1$) at -230 cm^{-1}, two additional peaks are observed. The strong peak at -300 cm^{-1} is assigned to ${}^{A}0^0_0$, which is always observed

and due to the collisional relaxation from $\widetilde{B}$ to $\widetilde{A}$ followed by the radiative decay. A very weak peak at -43 cm^{-1} is the decay to the low-frequency bending mode ν_2 (theo. 50 cm^{-1}). The similar spectra features of decays to the most-off diagonal Sr-O stretching mode and the low-frequency bending mode and the appearance of the ${}^{A}0_0^0$ peak when exciting to $\widetilde{B}$ have been observed for all other molecules. A more complex decay scenario is observed for SrOPh-3-CF_3 mainly due to the low-symmetric structure introduced by the electron-withdrawing group of CF_3. More vibrational decays have been observed. In Figure S1e, three fundamental vibrational modes have been resolved from the decay of $\widetilde{A}$ and assigned to ν_2, ν_5 and ν_6. The decay from the $\widetilde{B}$ state is more complicated due to the relaxation decay to the $\widetilde{A}$ state. Except for the same vibrational decays from $\widetilde{B}$, three more peaks are observed. The strongest peak at -300 cm^{-1} is due to the collisional relaxation and fluorescence pathways of $\widetilde{B} \rightarrow \widetilde{A} \rightarrow \widetilde{X}$. The high intensity is likely caused by the large CF_3 group which increases the collisional relaxation rate from the $\widetilde{B}$ state. Two other weak peaks are assigned to ${}^{A}5_0^1$ ${}^{A}6_0^1$ due to the relaxation from $\widetilde{B}$ to the vibrationally excited states of $\widetilde{A}$ followed by the fluorescence decays. The vibrational frequencies and VBRs of all observed vibrational modes are given in Tables S1 and S2. The radiative lifetimes of all excited states were measured to be $\approx 21 - 34$ ns, as shown in Figure S3, which are comparable to the lifetimes of CaOPh-X molecules.[8]

Error analysis of VBRs

All observed peaks in DLIF spectra in Figures 2 and S1 are fitted with the Gaussian function using the parameters of peak location, height and width. For the ith peak in each spectrum, the peak area (M_i) is extracted and the error of the area (δM_i) is estimated from the covariance matrix of the fitting parameters. The intensity ratios of each peak, as shown in Figure 3a, is obtained from the ratio of M_i and the total area of all the observed peaks, $\sum_{i=0}^{p} M_i$. The statistical error of each intensity ratio is then calculated from the relative uncertainties $\delta M_i/M_i$. Besides the statistical fitting errors, several sources of systematic

error in the DLIF measurement are discussed and listed in Table S3.

The first systematic error come from the unobserved peaks which contribute to the VBRs. A true VBR depends on contributions of all possible decay pathways. Due to a low measurement sensity and a small detection window, only a few vibrational decays have been observed for each transition. Compared to a complete description of vibrational decays obtained from calculated FCFs, all unobserved vibrational decays are therefore a source of the systematic uncertainty, which is estimated by:[8]

$$S_0' = \frac{S_0}{\sum_{i=0}^{p} S_i + \sum_{i=p+1}^{N} \frac{T_i}{C}}, \tag{2}$$

where S_0' is the scaled diagonal VBR considering contributions from the unobserved vibrational decays, S_0 is the observed intensity ratio of the diagonal peak, S_i is the observed intensity ratio of the ith vibational decay, T_i is the calculated VBR of the ith vibational decay and C is a scaling factor that averages the ratio of theoretical VBR (T_i) to experimental intensity ratio (S_i) for all observed peaks. Since the VBRs for bending modes are underestimated by theory, T_0 is usually larger than or roughly equal to S_0, thus the lowest scaled VBR S_0'' can be obtained when excluding the T_0/S_0 in the scaling factor and used as an estimate for the lower bound value of the diagonal VBR. For the measured intensity ratio S_0 is always overestimated and used as the upper bound of the diagonal VBR, while the scaled VBR S_0' is used as the plot data points in Figure 3. As summarized in Table S3, the uncertainties of the unobserved peaks as differences of S_0 and S_0'' are in the range of $1.1 - 3.2\%$.

Another source of systematic uncertainty is the signal drifting in the measurement due to the change of experimental conditions. Except for the DLIF measurment of SrOPh $\widetilde{B} \to \widetilde{X}$ by the cw laser and EMCCD (Figure S2), all other DLIF spectra were taken by scanning the grating of the spectrometer to disperse photons onto a PMT. This means that the fluorescence photons at different wavelengths were not detected simultaneously. A typical scan of 15 nm

wavelength range would take 75 minutes with an increment of 0.1 nm and 300 averages for each wavelength. During the scan, the signal was slowly drifting mainly due to the dust accumulation on the imaging lens and the pulse intensity drifting from both the ablation and the excitation lasers. We kept track of the signals before and after the whole scan and found that the signal change can vary by up to 20%, which can lead to an error of 1.0% in VBR estimation as such signal change mainly affects the off-diagonal vibrational transition signal. In addition, the dispersed photons at different wavelength were detected simultaneously in the EMCCD measurement, which eliminates the error due to signal drifting. The error can be estimated by the difference of the diagonal VBR of SrOPh $\widetilde{B} \to \widetilde{X}$ from the two different methods, which is 1.2 %.

As discussed in the error analysis of CaOPh-X,[8] the wavelength response of the spectrometer and the imperfection of the mirrors and lenses in the imaging system could cause a systematic error up to $\approx 1\%$. The last error source comes from the diagonal excitation of vibrationally excited modes in the ground state. Due to a cell temperature of ≈ 23 K, the thermalized molecules can have thermal populations of $\approx 5\%$ of the low-frequency bending mode and $\approx 10^{-6}$ of the stretching mode. Those vibrationally excited states in the $\widetilde{X}$ state could be near-resonantly excited by the pulsed dye laser to the same vibrational levels of the upper states. The following decays from those diagonal excitations can cause an error up to 0.5%.

By adding the four systematic errors in quadrature, a total systematic uncertainty is estimated to be $1.9\% - 3.5\%$. Considering the statistical uncertainties from the Gaussian fits, the final upper and lower bounds of uncertainties for the diagonal 0_0^0 VBRs are obtained and plotted in Figure 3b.

Fitting of high-resolution excitation spectrum of SrOPh

The rotationally-resolved excitation spectrum of SrOPh has been fitted to estimate the molecular constants. The energy levels of SrOPh $\widetilde{B}$ and $\widetilde{X}$ states are computed from an

effective Hamiltonian which includes rotation, electron spin-rotation coupling and centrifugal distortion correction terms:

$$H_{\text{eff}} = H_{\text{Rot}} + H_{\text{SR}} + H_{\text{cd}}. \tag{3}$$

The rotational Hamiltonian is

$$H_{\text{Rot}} = AN_a^2 + BN_b^2 + CN_c^2, \tag{4}$$

the spin-rotation coupling term is

$$H_{\text{SR}} = \epsilon_{aa}N_aS_a + \epsilon_{bb}N_bS_b + \epsilon_{cc}N_cS_c, \tag{5}$$

and the centrifugal distortion correction used in this work is

$$H_{\text{cd}} = -D_N N^2(N+1)^2 - D_{NK}N(N+1)K_a^2 - D_K K_a^4 + H_K K_a^6, \tag{6}$$

here the sextic centrifugal distortion correction H_K is included to enhance the fitting quality of the bandheads of the observed $\Delta K_a = \pm 1$ bands.[10]

Although a few rotational bands recognized in the SrOPh excitation spectrum can determine a few rotational constants in good accuracy, the high density of rotational line near 0-0 transition ($\sim 10^2$/GHz in the computing limit of $J_{max} = 30$) is the main challenge for fitting the rest of the parameters. Therefore, we used a homemade program first to search for parameters that can roughly fit the spectrum in contour and limit the searching range of the parameters for PGOPHER's subsequent fitting.[11] To avoid the program being trapped by local minima, two algorithms were used in turns: the mini-batch gradient descent (MBGD)[12] and the genetic algorithm (GA).[13] MBGD works similarly as the well-known gradient descent method, while its gradient is computed from a batch of randomly chosen data instead of the whole data set during each iteration to jump out of local minima with semi-stochastic steps. MBGD is the main algorithm that searches for a potential solution iteratively, and

GA checks whether the MBGD result is optimal within a larger parameter space. The target functions of the two algorithms are different, with the purpose of making their local minima to be also different.

A contour fitting result is accepted as the initial input of PGOPHER if it is agreed by both MBGD and GA. In PGOPHER, the fitting of molecular constants is done in following procedure: first, the clearly observed bandheads are matched to the simulated rotational bands, such as the lines labeled in Figure 4b and 4c. With such assignment PGOPHER can calculate parameters T_0, A, $\bar{B}$, ϵ_{aa}, D_K and H_K in a better accuracy than the contour fitting. Next, the rest of the parameters are obtained by some details of the spectrum near the 0-0 transition, such as the spacing of lines in different rotational bands, shape of peaks, and the order of line strength. For concreteness, some strong peaks in the middle of the spectrum (Figure S4a) are assigned to the transitions with $K_a'' = 0, 1, 2$ and different P, Q and R branches (P, Q, and R labels refer transitions with $\Delta J = -1, 0$ and 1, respectively): the strongest few peaks in Q branch are assigned to some observed peaks, see Figure S4b; and for the P and R branch transitions, the line assignment could be made according to some local features, see Figure S4c.

Fine adjustment of fitting is achieved by adjusting the parameters, re-assigning some lines or bands tentatively, and running the fit based on the updated assignment. This procedure should be repeated multiple times before the parameters become converged.

The rotational temperature in PGOPHER simulation is set based on the normalized strength of the rotational bandhead of $K_a' = 6 \leftarrow K_a'' = 5$, the farthest band we can recognize in experiment. It is found to be close to the experimental results when $T_{\rm sim}$ is around $2-3$ K. In the contour fitting procedure, the rotational temperature is estimated to be $4-7$ K, depending on the fitting condition such as linewidth and the upper limit of the rotational quantum number J_{max}.

The estimated molecular constants reported in Table 1 are from the best fit result whose simulated spectrum pattern matches most of observed peaks near the 0-0 transition, with the

assignment error bars calculated from the standard errors of the estimated values given by multiple fitting attempts. These attempts follow the same rotational bandhead assignment in the first step and have a similar P, Q, R line distributions depicted in Figure S4, while the numbers and the positions of assigned lines near the 0-0 transition are varied to reflect the parameter fluctuations caused by different assignments. During the fitting procedure, we noticed that the fitted values of the centrifugal distortion constants D_N, D_{NK} are very sensitive to the line assignment near the 0-0 transition, while their values could be determined if a set of rotational line assignment is given. In our best fit, PGOPHER reports that $D_{NK} = -2.8(5) \times 10^{-6}$ and $D_N = -1.4(5) \times 10^{-7}$ for the $\widetilde{X}$ state, and $D_{NK} = -5(2) \times 10^{-7}$ and $D_N = -1.4(5) \times 10^{-7}$ for the $\widetilde{B}$ state, respectively.

We also examined how much the quality of fitting would be changed if we fixed these centrifugal distortion constants to 0. Since the fitting in PGOPHER is based on the assigned rotational line positions, one straightforward way of comparing the fitting results is to compare the average error of these assigned lines, which is the average difference between the assigned and calculated rotational line frequencies. For our best fit, such average error is about 69 MHz; if D_N, D_{NK} in both X and B states are fixed to 0, the average error of the best fitting under such configuration is 73 MHz; if H_K is removed for both X and B states while keeping all the D_N and D_{NK} terms, the average error is 166 MHz. The parameter D_K in the X and B states are critical, and no reasonable fitting result could be obtained without them. Given that the linewidth in our fitting is set as 70 MHz and the step size of the scan is about 25-50 MHz, the average error of the assigned lines in our best fit is acceptable.

Spin-orbit coupling effect in SrOPh

According to the second order perturbation theory,[14,15] the relation between the SOC constant A_{so} and the effective spin-rotation constant of the $\widetilde{B}$ state could be estimated by the following equation:

$$\epsilon_{aa}^{\widetilde{B}} \approx -\frac{4AA_{\mathrm{so}}}{E_{\widetilde{B}} - E_{\widetilde{A}}}. \tag{7}$$

With the measured constants, the SOC constant in SrOPh estimated by Eq.(7) is $A_{\text{so}} \approx 272\text{cm}^{-1}$, which is similar to the A_{so} of the SrOH $A^2\Pi$ state[16] (≈ 265 cm^{-1}).

The SO interaction can also explain why the energy gaps between $\widetilde{A}$ and $\widetilde{B}$ states (≈ 300 cm^{-1}) of all the species shown in Figure 1b are much larger than their prediction. In the calculation, the electronic energies are computed without SOC effect, and the excited state wavefunctions, conventionally labeled as $|A^2B_2\rangle$ and $|B^2B_1\rangle$, are assumed to follow the C_{2v} symmetry. However, the SO interaction strongly mixes the two states and breaks the C_{2v} symmetry in these wavefunctions. We can use a simple two level system model to demonstrate such mixing, in which the total electronic Hamiltonian is contributed by the electronic Hamiltonian ($H_{\text{el}} = E_A|A^2B_2\rangle\langle A^2B_2| + E_B|B^2B_1\rangle\langle B^2B_1|$) and the SO interaction Hamiltonian:

$$H_{\text{tot}} = H_{\text{el}} + H_{\text{so}}, \tag{8}$$

here the SO coupling Hamiltonian is $H_{\text{SO}} = A_{\text{so}}L_aS_a$, L_a and S_a are the projection operators of the orbital and spin angular momentum onto the principle axis a, respectively. As written in the basis of the electronic states $\{|A^2B_2\rangle, |B^2B_1\rangle\}$, H_{tot} reads:[17]

$$H_{\text{tot}} = \begin{pmatrix} E_A & A_{\text{so}}\Sigma \\ A_{\text{so}}\Sigma & E_B \end{pmatrix} = \frac{E_B + E_A}{2} + \frac{1}{2}\begin{pmatrix} -\Delta E_0 & A_{\text{so}} \\ A_{\text{so}} & \Delta E_0 \end{pmatrix}, \tag{9}$$

where $\Delta E_0 = E_B - E_A$ is the energy separation between the $|A^2B_2\rangle$ and $|B^2B_1\rangle$ states without the SO interaction, and it could be regarded as the splitting caused by the molecular asymmetry. Σ is the projection quantum number of electronic spin onto the principle axis a, and we take $\Sigma = \frac{1}{2}$ since its sign does not affect the energy levels. According to Eq.(9), the energy difference between the two eigenstates of H_{tot}, $\widetilde{A}$ and $\widetilde{B}$, could be calculated by the following equation:

$$\Delta E = E_{\widetilde{B}} - E_{\widetilde{A}} = \sqrt{(\Delta E_0)^2 + A_{\text{so}}^2}. \tag{10}$$

For SrOPh, the total energy gap is $\Delta E = 305$ cm^{-1}, which then gives $\Delta E_0 = 138$ cm^{-1} $\ll$

A_{so}. This implies that the orbital angular momentum is partially preserved by the strong SO interaction in the SrOPh $\widetilde{A}$ ($\widetilde{B}$) state. Therefore, the SrOPh $\widetilde{A}$ ($\widetilde{B}$) state is similar to a $^2\Pi_{|\Omega|=\frac{1}{2}}$($^2\Pi_{|\Omega|=\frac{3}{2}}$) state in the symmetric top approximation. Noticed that ΔE is also much larger than ΔE_0 in the other species studied in this work, the SO interaction is expected to dominate the separation between the $\widetilde{A}$ and $\widetilde{B}$ states of all these species.

References

(1) Frisch, M. J.; Trucks, G. W.; Schlegel, H. B.; Scuseria, G. E.; Robb, M. A.; Cheeseman, J. R.; Scalmani, G.; Barone, V.; Petersson, G. A.; Nakatsuji, H. et al. Gaussian 16 Revision C.01. 2016; Gaussian Inc. Wallingford CT.

(2) Perdew, J. P.; Ernzerhof, M.; Burke, K. Rationale for Mixing Exact Exchange with Density Functional Fpproximations. *J. Chem. Phys.* **1996**, *105*, 9982–9985.

(3) Weigend, F.; Ahlrichs, R. Balanced Basis Sets of Split Valence, Triple Zeta Valence and Quadruple Zeta Valence Quality for H to Rn: Design and Assessment of Accuracy. *Phys. Chem. Chem. Phys.* **2005**, *7*, 3297–3305.

(4) Grimme, S.; Antony, J.; Ehrlich, S.; Krieg, H. A Consistent and Accurate ab initio Parametrization of Density Functional Dispersion Correction (DFT-D) for the 94 Elements H-Pu. *J. Chem. Phys.* **2010**, *132*, 154104.

(5) Rappoport, D.; Furche, F. Property-Optimized Gaussian Basis Sets for Molecular Response Calculations. *J. Chem. Phys.* **2010**, *133*, 134105.

(6) Lu, T.; Chen, F. Multiwfn: a Multifunctional Wavefunction Analyzer. *J. Comput. Chem.* **2012**, *33*, 580–592.

(7) Dickerson, C. E.; Guo, H.; Shin, A. J.; Augenbraun, B. L.; Caram, J. R.; Camp-

bell, W. C.; Alexandrova, A. N. Franck-Condon Tuning of Optical Cycling Centers by Organic Functionalization. *Phys. Rev. Lett.* **2021**, *126*, 123002.

(8) Zhu, G.-Z.; Mitra, D.; Augenbraun, B. L.; Dickerson, C. E.; Frim, M. J.; Lao, G.; Lasner, Z. D.; Alexandrova, A. N.; Campbell, W. C.; Caram, J. R. et al. Functionalizing Aromatic Compounds with Optical Cycling Centers. *Nature Chem.* **2022**, *14*, 995–999.

(9) Augenbraun, B. L.; Doyle, J. M.; Zelevinsky, T.; Kozyryev, I. Molecular Asymmetry and Optical Cycling: Laser Cooling Asymmetric Top Molecules. *Phys. Rev. X* **2020**, *10*, 031022.

(10) Brazier, C. R.; Bernath, P. F. High-Resolution Laser Spectroscopy of the $\widetilde{A}\,^2B_2 - \widetilde{X}\,^2A_1$ and $\widetilde{B}\,^2B_1 - \widetilde{X}\,^2A_1$ Systems of $SrNH_2$. *J. Mol. Spectrosc.* **2000**, *201*, 116–123.

(11) Western, C. M. PGOPHER: A Program for Simulating Rotational, Vibrational and Electronic Spectra. *J. Quant. Spectrosc. Radiat. Transf.* **2017**, *186*, 221–242.

(12) Konečnỳ, J.; Liu, J.; Richtárik, P.; Takáč, M. Mini-Batch Semi-Stochastic Gradient Descent in the Proximal Setting. *IEEE J. Sel. Top. Signal Process.* **2015**, *10*, 242–255.

(13) Meerts, W. L.; Schmitt, M.; Groenenboom, G. C. New Applications of the Genetic Algorithm for the Interpretation of High-Resolution Spectra. *Can. J. Chem.* **2004**, *82*, 804–819.

(14) Van Vleck, J. H. The Coupling of Angular Momentum Vectors in Molecules. *Rev. Mod. Phys.* **1951**, *23*, 213.

(15) Morbi, Z.; Zhao, C.; Bernath, P. F. A High-Resolution Analysis of the $\widetilde{C}\,^2A_1 - \widetilde{X}\,^2A_1$ Transition of $CaNH_2$: Pure Precession in Polyatomic Molecules. *J. Chem. Phys.* **1997**, *106*, 4860–4868.

(16) Presunka, P. I.; Coxon, J. A. Laser Excitation and Dispersed Fluorescence Investigations of the $\widetilde{A}\,^2\Pi - \widetilde{X}\,^2\Sigma^+$ System of SrOH. *Chem. Phys.* **1995**, *190*, 97–111.

(17) Liu, J. Rotational and Fine Structure of Open-Shell Molecules in Nearly Degenerate Electronic States. *J. Chem. Phys.* **2018**, *148*, 124112.

(18) Lim, I. S.; Stoll, H.; Schwerdtfeger, P. Relativistic Small-Core Energy-Consistent Pseudopotentials for the Alkaline-Earth Elements from Ca to Ra. *J. Chem. Phys.* **2006**, *124*, 034107.

Table S1: Comparison of the observed and calculated frequencies for resolved fundamental vibrational modes of all species studied in this work. Values are given in units of cm^{-1}.

Vib. modes	SrOPh		Vib. modes	$SrOPh\text{-}3\text{-}CF_3$	
	Exp.	Theo.		Exp.	Theo.
ν_2	54(2)	56	ν_2	42(5)	39
$2\nu_2$	102(2)	112	ν_5	178(5)	180
ν_3	238(2)	241	ν_6	219(2)	222
$\nu_2\nu_3$	297(2)	297			
Vib. modes	SrOPh-3-F		Vib. modes	$SrOPh\text{-}3\text{-}CH_3$	
	Exp.	Theo.		Exp.	Theo.
ν_1	56(5)	56	ν_2	43(5)	50
ν_3	226(2)	222	ν_5	226(2)	230
Vib. modes	$SrOPh\text{-}3,4,5\text{-}F_3$				
	Exp.	Theo.			
ν_2	47(6)	45			
ν_4	203(2)	204			

Table S2: The intensity ratios of all observed vibrational decays of all molecules. The errors indicate the statistical uncertainties from the Gaussian fits. The theoretical VBRs are also added for comparison.

Modes	$SrOPh\text{-}3\text{-}CH_3$			
	Exp.(A)	Theo.(A)	Exp.(B)	Theo.(B)
0	0.946(6)	0.926	0.918(9)	0.943
ν_2		7×10^{-4}	0.011(2)	2×10^{-4}
ν_5	0.054(6)	0.059	0.072(9)	0.044
Modes	SrOPh			
	Exp.(A)	Theo.(A)	Exp.(B)	Theo.(B)
0	0.845(7)	0.928	0.885(5)	0.945
ν_2	0.029(3)	$< 10^{-4}$	0.054(3)	$< 10^{-4}$
$2\nu_2$	0.025(3)	$< 10^{-4}$		
ν_3	0.079(5)	0.059	0.060(3)	0.043
$\nu_2\nu_3$	0.021(3)	$< 10^{-4}$		
Modes	SrOPh-3-F			
	Exp.(A)	Theo.(A)	Exp.(B)	Theo.(B)
0	0.956(13)	0.936	0.965(3)	0.954
ν_1	0.007(3)	9×10^{-4}		
ν_3	0.037(16)	0.051	0.035(3)	0.037
Modes	$SrOPh\text{-}3\text{-}CF_3$			
	Exp.(A)	Theo.(A)	Exp.(B)	Theo.(B)
0	0.893(5)	0.939	0.867(11)	0.950
ν_2	0.014(2)	0.003	0.021(5)	$< 10^{-4}$
ν_5	0.018(2)	0.007	0.026(6)	0.007
ν_6	0.074(3)	0.037	0.086(8)	0.035
Modes	$SrOPh\text{-}3,4,5\text{-}F_3$			
	Exp.(A)	Theo.(A)	Exp.(B)	Theo.(B)
0	0.946(7)	0.945	0.964(4)	0.963
ν_1	0.005(2)	$< 10^{-4}$	0.008(2)	$< 10^{-4}$
ν_4	0.049(7)	0.044	0.028(4)	0.030

Table S3: Systematic error budget for the vibrational branching ratio measurements.

VBRs measurement error source	Percentage
Contributions from unobserved peaks	1.1% – 3.2%
Signal fluctuation	1.0%
Instrument wavelength response	1.0%
Diagonal excitation	0.5%
Total error	1.9% – 3.5%

Table S4: Measured intensity ratios and scaled VBRs of the diagonal 0-0 decay of all molecules. The scaling process considering contributions of unobserved vibrational decays is detailed in the section of error analysis of VBRs.

$\widetilde{A} \to \widetilde{X}$ Transition					
Molecules	Measured VBR S_0	Scaled VBR S_0'	Scaled exluding main peak S_0''	Upper error bar	Lower error bar
SrOPh-3-CH_3	0.946(6)	0.933	0.933	0.015	0.006
SrOPh	0.845(7)	0.822	0.804	0.024	0.020
SrOPh-3-F	0.956(13)	0.943	0.941	0.019	0.013
SrOPh-3-CF_3	0.893(5)	0.868	0.857	0.026	0.012
SrOPh-3,4,5-F_3	0.946(7)	0.930	0.924	0.017	0.009
$\widetilde{B} \to \widetilde{X}$ Transition					
Molecules	Measured VBR S_0	Scaled VBR S_0'	Scaled exluding main peak S_0''	Upper error bar	Lower error bar
SrOPh-3-CH_3	0.918(9)	0.896	0.880	0.023	0.019
SrOPh	0.885(5)	0.869	0.859	0.017	0.012
SrOPh-3-F	0.965(3)	0.958	0.958	0.008	0.003
SrOPh-3-CF_3	0.867(11)	0.853	0.840	0.018	0.017
SrOPh-3,4,5-F_3	0.964(4)	0.953	0.950	0.011	0.005

Table S5: Vertical excitation energies and Franck-Condon factors of SrOPh and SrOPh-3,4,5-F_3 computed using various functionals and ECP28MWB relativistic effective core potential (ECP) with the def2-TZVPPD basis set.[18]

Functional	FCF_{00} of $\widetilde{A} \to \widetilde{X}$	Vertical excitation of $\widetilde{A} \leftarrow \widetilde{X}$ (eV)	Vertical excitation of $\widetilde{B} \leftarrow \widetilde{X}$ (eV)
SrOPh			
PBE0-D3, ECP28MWB	0.9323	1.7787	1.7871
wB97XD, ECP28MWB	0.8991	1.6994	1.7085
SrOPh-3,4,5-F_3			
PBE0-D3, ECP28MWB	0.948	1.8030	1.8085
wB97XD, ECP28MWB	0.919	1.7261	1.7336

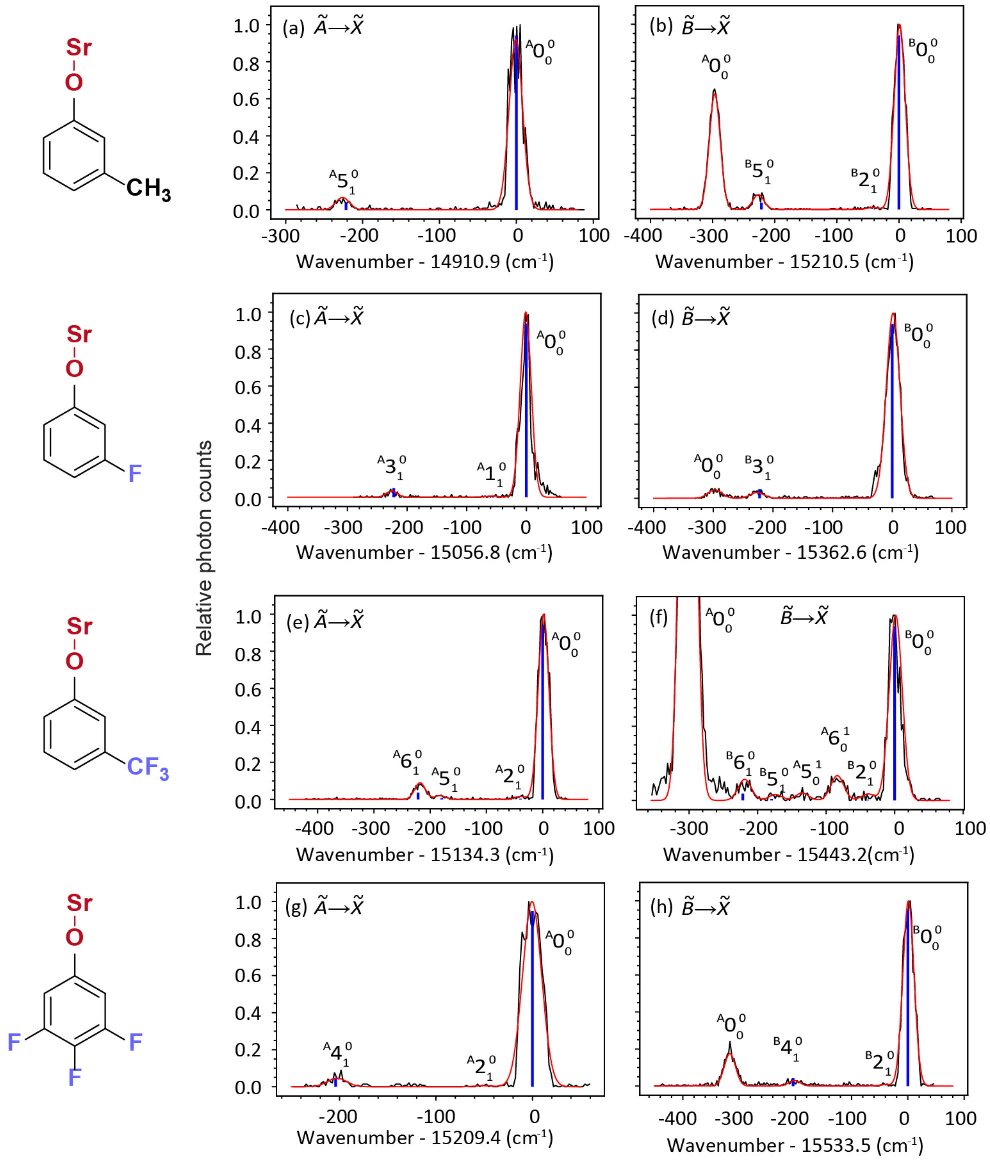

Figure S1: Dispersed fluorescence spectra of all species. The experimental curves (black) are fitted with the Gaussian functions (red). The blue sticks illustrate the vibrational branching ratios of different vibrational modes. The assignments of resolved vibrational peaks are also given.

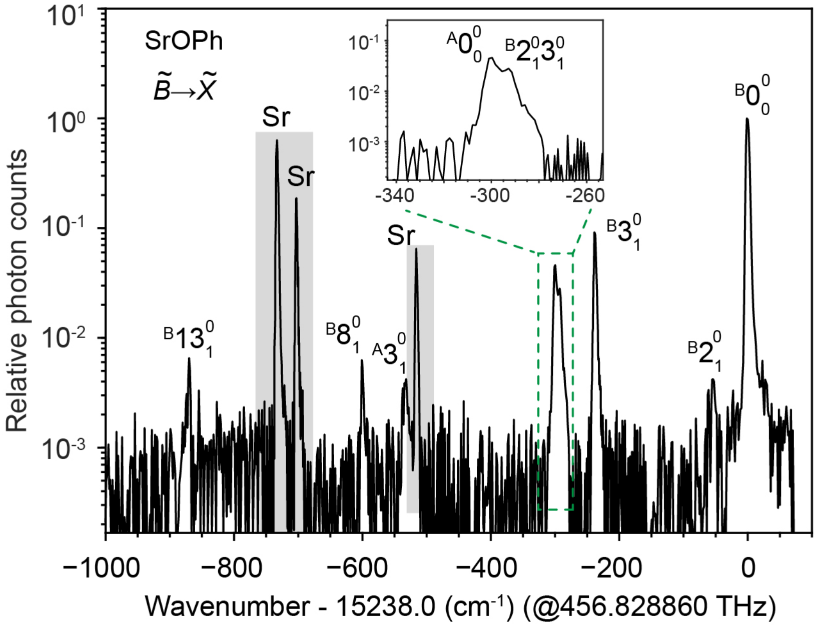

Figure S2: Dispersed spectrum of SrOPh $\widetilde{B} \rightarrow \widetilde{X}$ excited by cw laser and measured by a spectrometer coupled with an EMCCD camera. The inset shows the expansion of a broad peak at -300 cm^{-1}, which is due to the overlapping of two peaks. The assignments of the resolved vibrational peaks are also given.

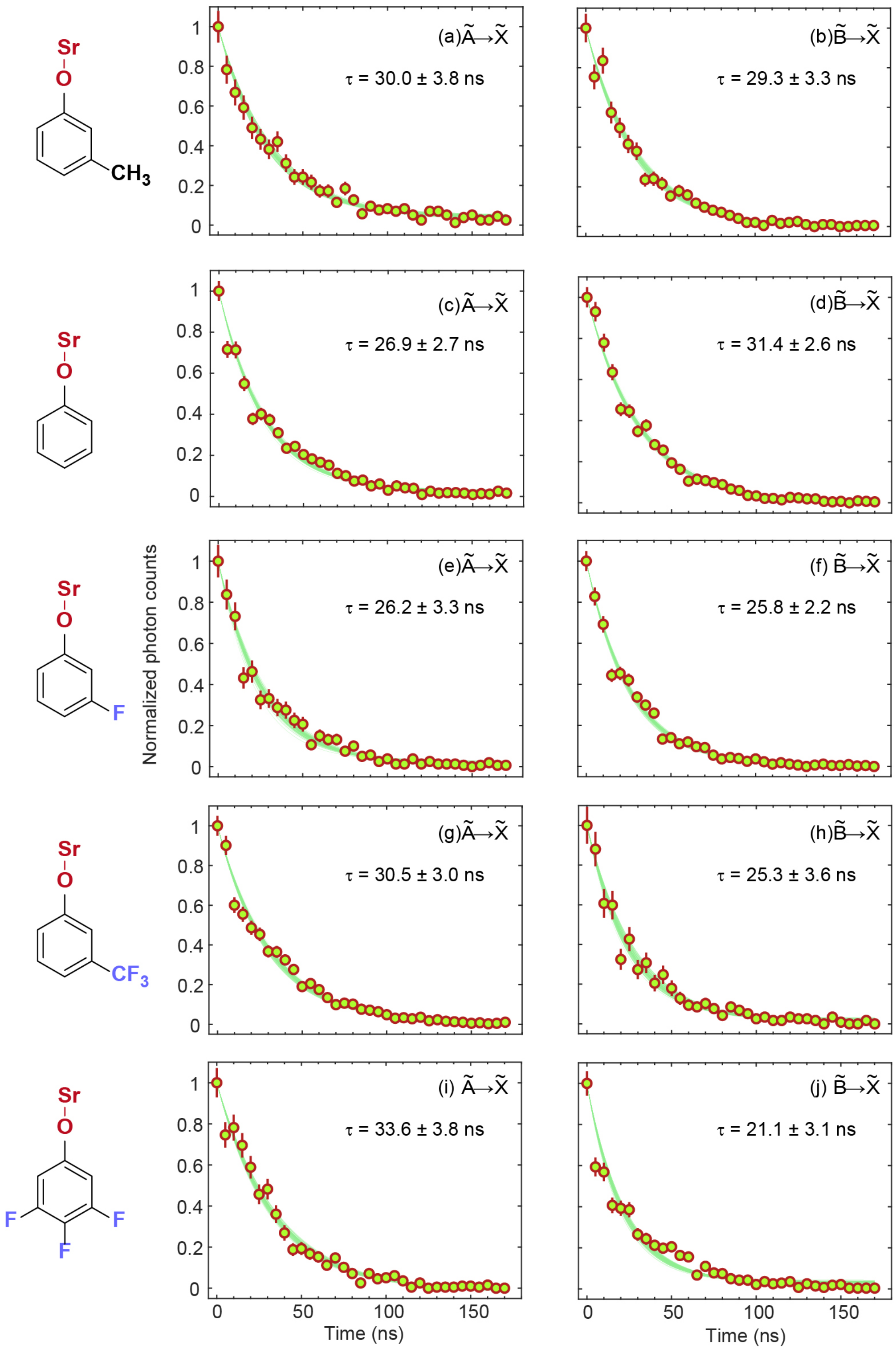

Figure S3: Fluorescence decay traces of all the transitions studied in this work. The experimental data points (red circles) are obtained from the sum of PMT signal for the $0-0$ decay in the DLIF measurements and the respective error bar represents the standard errors. For each trace, the data points are normalized to the maximum signal counts. The radiative lifetimes τ and errors for all species are estimated from exponential fits (green curves) by bootstrapping the data.

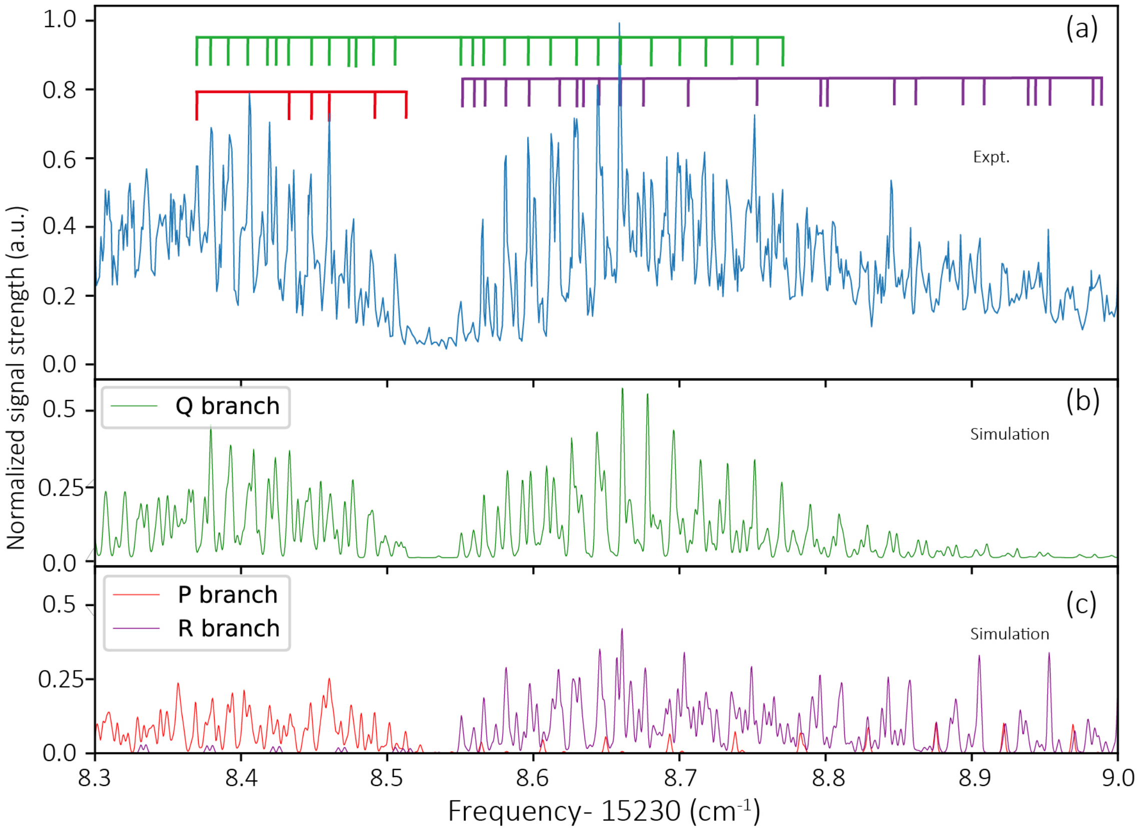

Figure S4: A typical line assignment near 0-0 transition of SrOPh. The experimental (a) and simulated data (b,c) are normalized to the maximum signal strength in experiment and simulation, respectively. The observed peaks labeled with green, purple and red ticks in the (a) measured spectrum are assigned to the simulation peaks of (b) Q, (c) R and P branch transitions, respectively. The Gaussian linewidth for the simulation is set as 70 MHz to roughly fit the contour, with the rotational temperature set as $T_{\text{sim}} = 2.5$ K. Each simulation peak in this range usually contains multiple rotational lines, to avoid overfitting, only the strongest 2 - 4 lines in each peak are assigned to the corresponding observed peak.
