# Supplementary material for: Laser Spectroscopy of Aromatic Molecules with Optical Cycling Centers: Strontium(I) Phenoxides
Source: J Phys Chem Lett. 2022 Nov 22;13(47):11029–35. doi: 10.1021/acs.jpclett.2c03040 (PMC9720742; doi:10.1021/acs.jpclett.2c03040)
Supplement: Supplementary file 2 — jz2c03040_si_002.pdf [file jz2c03040_si_002.pdf]

jz-2022-03040w.R1

Name: Peer Review Information for "Laser Spectroscopy of Aromatic Molecules with Optical Cycling Centers: Strontium (I) Phenoxides"

First Round of Reviewer Comments

Reviewer: 1

Comments to the Author
Following their computational and spectroscopic studies on the CaOR molecules with optical cycling centers (OCCs), the authors report their investigations of SrOR molecules in the reviewed manuscript. This is a combined theoretical/computational and experimental study. Both vibronically and rotationally resolved (dispersed) laser-induced fluorescence spectra have been obtained and analyzed. The experimental spectra are of high quality in terms of both resolution and signal-to-noise ratio. The simulation and fitting of the high-resolution LIF spectrum are solid and convincing. Some of the target molecules may be good candidates for direct laser-cooling and have broad applications in many research fields. I strongly support the publication of this manuscript in the J. Phys. Chem. Lett.

Almost the only scientific issue that concerns me is fitting the high-resolution LIF spectrum. Table 1. Their error bars of centrifugal distortions constants are comparable or, in some cases, larger than their absolute values. It is not very clear to me why the higher-order centrifuge distortion constant H_K needs to be included in the fitting. How much does the standard deviation (rms) of the fitting increase when these CD constants are fixed to zero?

The dominant contribution to ϵ_{bb} and ϵ_{cc} of the B state comes from the interaction between the C and B states. Therefore, an approximate relation: $\epsilon_{bb} / \epsilon_{cc} \approxeq B / C$ is expected. I don't know whether one can impose such a restriction in PGOPHEr when running the spectral fitting. If yes, I think it is worth trying.

A comment on VBR: If
(1) one neglects the interactions between the C state and the A/B state, i.e., one regards the X, A, and B states as an isolated system, and
(2) the A and B states are considered together (as one electronic state),
the Frank-Condon constants for the origin bands of the excitation transition [B/A<--X (0, 0)] and the emission transition [B/A-->X (0, 0)] are supposed to be the same. I think it would be easier to obtain an accurate value for f_{00} in the LIF experiment than the DF.

Reviewer: 2

Comments to the Author
This is a nice paper presenting combined theory and experiment efforts aiming

to laser-cool large organic molecules. The authors describe Bz-O-Sr molecules with electron-withdrawing groups.

Overall, this is an important contribution and should be published, however, there are several important shortcomings that need to be addressed:
1. Functionlalized organic molecules and their potential for laser cooling has been discussed in this paper, which reported high level theoretical treatment of their electronic structure and FCFs:
J. Phys. Chem. Lett. 11, 6670 – 6676 (2020)
These paper (together with Ref. 33) reported the results for functionalized benzene -- with one or 2 OCCs quite before authors own work.

In addition, another theoretical study has investigated the impact of the electron-withdrawing ability of the linker on FCFs in somewhat simpler molecules, M-X, where X=H,OH, CCH, NCO, NC, OBO: Phys. Chem. Chem. Phys. 21, 19447 – 19457 (2019).
This study also needs to be mentioned when the authors discuss the idea of using electronegativity as one of the control knobs that can tune the FCFs.

2. The theoretical methods are not described with sufficient details, it is not even clear how FCFs were computed. I recommend to expand this part in the SI. I also have a concern about electronic structure calculations. The failure of standard functionals to treat Rydberg states (in TDDFT) is well known -- they are affected by self-interaction error. These molecules are of Rydberg character. How did the authors validated their choice of the DFT protocol? This needs to be mentioned in the main text.

3. Finally, this sentence needs to be corrected:
``The somewhat unexpected atom-like transitions supporting optical cycling and cooling in these small molecules have inspired searches for similar transitions in complex polyatomic molecules with an M-O-R structure,10 where M is an alkaline-earth metal atom ionically bonded to oxygen (O) forming an optical cycling center (OCC) and R is a molecular ligand.33–35''

The authors should directly acknowledge the pioneering work of Isaev andd Berger (ref. 9 in the current draft), who were the first to identify this promising motif for the design of OCCs in polyatomic molecules, on the basis of molecular orbital theory. Their insight was quite remarkable and their proposal remains to be the most commonly utilized in the field.

Author's Response to Peer Review Comments:

Dear Editor,

We thank both you and the reviewers for the careful consideration of our manuscript. We have made the non-scientific changes for references, title and headers as editors suggested. A new colored Scheme 1 and a separate supporting information file are provided. In what follows, we have provided a detailed response to each of the referees’ comments and suggestions.

Sincerely,
Guo-Zhu Zhu

Referee 1

Following their computational and spectroscopic studies on the CaOR molecules with optical cycling centers (OCCs), the authors report their investigations of SrOR molecules in the reviewed manuscript. This is a combined theoretical/computational and experimental study. Both vibronically and rotationally resolved (dispersed) laser-induced fluorescence spectra have been obtained and analyzed. The experimental spectra are of high quality in terms of both resolution and signal-to-noise ratio. The simulation and fitting of the high-resolution LIF spectrum are solid and convincing. Some of the target molecules may be good candidates for direct laser-cooling and have broad applications in many research fields. I strongly support the publication of this manuscript in the J. Phys. Chem. Lett.

Almost the only scientific issue that concerns me is fitting the high-resolution LIF spectrum. Table 1. Their error bars of centrifugal distortions constants are comparable or, in some cases, larger than their absolute values. It is not very clear to me why the higher-order centrifuge distortion constant H_K needs to be included in the fitting. How much does the standard deviation (rms) of the fitting increase when these CD constants are fixed to zero?

The inclusion of the higher-order centrifuge distortion (CD) constant H_K is to improve the line assignments. We have spent some time on fitting without the constant H_K, but we found that such a configuration could not fit the bandheads belonging to the $K’’>4$ rotational states properly. To convince ourselves about the fitted values of H_K, we looked at the literature for papers on the spectroscopy of asymmetric top molecules. We found that, in the high-resolution laser spectroscopy study of SrNH2 [C. R. Brazier and P.F. Bernath, J. Mol. Spectros. 201, 116–123 (2000)], the H_K of SrNH2 X and A states were measured to be around -1.6x10^-6 and -2x10^-5 cm-1, respectively, which provides some support to our fit results in the aspect of the order of magnitude.

The error bars of CD constants are from the standard deviation of the fitted values obtained from several fitting attempts, with each of them having an individual line assignment in PGOPHER. Such error bars are usually one order of magnitude greater than the standard deviation given by the PGOPHER because the line assignment near the 0-0 transition is the main source of the fitting error in our case. During the fitting procedure, we noticed that the fitted values of the centrifugal distortion constants D_N, D_NK are very sensitive to the line assignment, while their values could be determined when a set of line assignment is given. In our best fit, PGOPHER gives D_NK=-2.8(5)x10^-6 and D_N=-1.4(5)x10^-7 for the X state, and D_NK =-5(2)x10^-6 and D_N =-1.4(5)x10^-7 for the B state, respectively. The removal of these constants might induce mismatches of some rotational lines.

We also examined how much the quality of fitting would be changed if we fixed these centrifugal distortion constants to 0. Since the fitting in PGOPHER is based on the assigned rotational line positions, one straightforward way of comparing the fitting results is to compare the average error of these assigned lines, which is the average difference between the assigned and calculated rotational line frequencies. For our best fit, such average error is about 69 MHz; if D_N, D_NK in both X and B states are fixed to 0, the average error of the best fitting under such configuration is 73MHz; if H_K is removed for both X and B states while keeping all the D_N and D_NK terms, the average error is 166 MHz. The parameter D_K in the X and B states are critical, and no reasonable fitting result could be obtained without them. Given that the linewidth in our fitting is set as 70 MHz and the step size of the scan is about 25-50 MHz, the average error of the assigned lines in our best fit is acceptable.

The additional explanations have been added and highlighted in the subsection of 'Fitting of high-resolution excitation spectrum of SOPh' in SI.

The dominant contribution to ϵ_{bb} and ϵ_{cc} of the B state comes from the interaction between the C and B states. Therefore, an approximate relation: $\epsilon_{bb} / \epsilon_{cc} \approxeq B / C$ is expected. I don't know whether one can impose such a restriction in PGOPHEr when running the spectral fitting. If yes, I think it is worth trying.

Following the referee's suggestion, we set one of the spin-rotation coupling constant fixed during each fitting iteration (ϵ_{cc} , for example), and then manually updated it with equation $\epsilon_{cc}\approx \epsilon_{bb}\times C/B$ before executing the next iteration. We tried running the fit for about 50-100 iterations and even re-assigned some lines to fit the contour near the 0-0 transition better, but we still haven't noticed any significant improvement in the fitting.

A comment on VBR: If
(1) one neglects the interactions between the C state and the A/B state, i.e., one regards the X, A, and B states as an isolated system, and
(2) the A and B states are considered together (as one electronic state),
the Frank-Condon constants for the origin bands of the excitation transition [B/A<--X (0, 0)] and the emission transition [B/A-->X (0, 0)] are supposed to be the same. I think it would be easier to obtain an accurate value for f_{00} in the LIF experiment than the DF.

We agree that the FCFs for the excitation transition [B/A<--X (0, 0)] and the emission transition [B/A<--X (0, 0)] are the same. The advantage of the dispersed fluorescence spectroscopy is to obtain the vibrational decay channels and their relative intensities from B/A (v'=0), which allows us to obtain the VBRs while only exciting along a single line without calibrating the laser intensity.

Referee 2

This is a nice paper presenting combined theory and experiment efforts aiming to laser-cool large organic molecules. The authors describe Bz-O-Sr molecules with electron-withdrawing groups.

Overall, this is an important contribution and should be published, however, there are several important shortcomings that need to be addressed:

1. Functionlalized organic molecules and their potential for laser cooling has been discussed in this paper, which reported high level theoretical treatment of their electronic structure and FCFs: J. Phys. Chem. Lett. 11, 6670 – 6676 (2020)
These paper (together with Ref. 33) reported the results for functionalized benzene -- with one or 2 OCCs quite before authors own work.

In addition, another theoretical study has investigated the impact of the electron-withdrawing ability of the linker on FCFs in somewhat simpler molecules, M-X, where X=H,OH, CCH, NCO, NC, OBO: Phys. Chem. Chem. Phys. 21, 19447 – 19457 (2019). This study also needs to be mentioned when the authors discuss the idea of using electronegativity as one of the control knobs that can tune the FCFs.

Thanks the referee for pointing out the references omission. We have added both references.

2. The theoretical methods are not described with sufficient details, it is not even clear how FCFs were computed. I recommend to expand this part in the SI. I also have a concern about electronic structure calculations. The failure of standard functionals to treat Rydberg states (in TDDFT) is well known -- they are affected by self-interaction error. These molecules are of Rydberg character. How did the authors validated their choice of the DFT protocol? This needs to be mentioned in the main text.

We thank the referee for their comments, we agree the theoretical details could be provided in more detail in the main text. We note the paragraph in the SI has details on how FCFs were calculated, with additional clarifying sentences we added in bold:

"Molecular geometries, excitation energies, and Franck Condon factor (FCF) calculations were performed in Gaussian16 [1]. ***Density functional theory (DFT) was used for the ground states while time-dependent DFT with 6 roots in the Davidson algorithm search space was used for excited states. A superfine grid and very tight convergence parameters were used to optimize geometries with the PBE0-D3 functional (with dispersion corrections) and the def2-TZVPPD basis set*** *[2–5]. An effective core potential (ECP) was used for the Sr atom within the def2-TZVPPD basis set.*

…The FCFs were calculated between the ground and first few low-lying excited electronic states, within the Condon and Born-Oppenheimer approximations. The FCFs were computed within the harmonic approximation and included Duschinsky rotations, which appeared to be sufficient for an overall trend in FCF. *However, the anharmonicity, higher-order effects such as Hertzberg-Teller coupling and other vibronic coupling effects do play a key role in the FCFs of the low-frequency bending modes, which were underestimated here in comparison to the experimental measurements."*

Additionally, we agree TD-DFT is poor for Rydberg excitations. However, this SrOPh electronic transition is a localized excitation from strontium's 5s to 5p/4d valence orbitals, instead of Rydberg-like excitations which involve higher (n+1)s or (n+1)p orbitals. We note that the radical is the remaining, unused valency of Sr, the other being consumed for the ionic bond with the ligand; hence, it is chemically not a Rydberg state, but rather a valence state. We quantify this

by running a state-selective ground state CASSCF CAS(3,6) calculation and find the unpaired electron has an orbital composition of: Sr: 3s (-0.25820), 4s (-0.28041), 5s(0.77481), and 6s (0.52169).

To quantify Rydberg excitation character, we look at the 2nd order moment expectation value of the 1-RDM matrix of excited states relative to ground state in CASSCF (Table 1). Rydberg states usually have larger 2nd order moments, where QMRR = the isotropic quadrupole moment and RR = XX+YY+ZZ, (2-3x larger) than the non-Rydberg valence state, and we note here it is only ~1.3 larger. [1,2]. Therefore, although there is some Rydberg character, it is dominated by the local valence electronic transition.

We performed a complete active space self-consistent field (CASSCF) calculation with 3 electrons and 6 orbitals (HOMO-1, HOMO, LUMO, LUMO+1, LUMO+2, LUMO+3) in the active space in Molpro [3]. The first and second excited states were computed with a state-averaged CAS calculation with weights (0.1, 0.8, 0.1) and (0.1, 0.1, 0.8), respectively. Additionally, we computed the 1-RDM (reduced density matrices) for all states to quantify the spatial extent of the wavefunction, labeled as <State|QMRR|State>.

**Electronic State**	**<State|QMRR|State>**
1	-80.938132884185
2	-95.743933618316
3	-105.982715836966

Table 1. 1-RDM 2nd-order moment values from a CAS(3,6) calculation of SrOPh with the def2-TZVPPD basis set and Stuttgart ECP28MWB on the Sr atom using DFT-optimized geometries.

Our methods were benchmarked on smaller molecules SrOH, SrOCH3, CaOH, CaOCH3 and compared to MRCI and experiment [4 (SI)]. Additionally, we have compared our DFT level of theory to experiment in previous papers for species such as CaOPh, and even larger arenes such as CaO-coronene [5, 6, 7 (SI)].

However, we agree some functional benchmarking could be done for SrOPh, and have performed calculations with two functionals (PBE0, and long-range corrected ωB97XD) with relativistic (Stuttgart) ECPs [8]. We have added the following benchmarking table and sentence to the SI:

"As seen in Table S5, the long-range corrected functional predicts more accurate FCFs and excitation energies, however, PBE0-D3 is sufficient in predicting FCF trends, as both predict the same relative changes in excitation energy (~0.03 eV) and FCF increase (0.020 for ωB97XD and 0.016 for PBE0-D3) from SrOPh and SrOPh-3,4,5-F_3."

Functional	FCF_00 of A->X	Vertical excitation of A<-X (eV)	Vertical Excitation of B<-X (eV)
SrOPh			
PBE0-D3, ECP28MWB	0.9323	1.7787	1.7871
ωB97XD, ECP28MWB	0.8991	1.6994	1.7085
SrOPh-3,4,5-F_3			
PBE0-D3, ECP28MWB	0.948	1.8030	1.8085
ωB97XD, ECP28MWB	0.919	1.7261	1.7336

Table S5. Vertical excitation energies and Franck-Condon factors of SrOPh and SrOPh-3,4,5-F_3 computed using various functionals and ECP28MWB relativistic effective core potential (ECP) with the def2-TZVPPD basis set. [8]

The persisting TD-DFT error across functionals suggests these functionals still lack important dynamic correlation. Valence states usually require larger dynamic correlation corrections than Rydberg states. Therefore, using methods which can improve upon correlation systematically, such as equation-of-motion coupled-cluster, should be investigated in future studies with these types of molecules, as demonstrated by previous work [9]. Additionally, methods which can include spin-orbit coupling, especially between the A and B states, would also improve theoretical predictions.

We have added a sentence in the main text to validate our DFT protocol:
"Due to the predominantly localized excitations and previous benchmarking results [4, 5, 6, 7, SI], we find TD-DFT sufficient to predict VBR trends in SrOPh optical cycling species. However, we find our theoretical calculations still lack important dynamic correlation and spin-orbit coupling which will affect important branching pathways. For high-level predictions beyond simple trends, we suggest choosing methods which can improve upon dynamic correlation systematically, such as coupled-cluster [9-11], and incorporating the Breit-Pauli operator to compute spin-orbit coupling effects [12]."

References:

1. Dong, S. S.; Gagliardi, L.; Truhlar, D. G. *J. Chem. Theory Comput.* **2019**, *15* (8), 4591–4601.

2. Love, D. E.; Nachtigallova, D.; Jordan, K. D.; Lawson, J. M.; Paddon-Row, M. N. *J. Am. Chem. Soc.* **1996**, *118* (6), 1235–1240.
3. MOLPRO, version , a package of ab initio programs, H.-J. Werner, P. J. Knowles, G. Knizia, F. R. Manby, M. Schütz, and others, see https://www.molpro.net.
4. Dickerson, C. E. et. al. *Phys. Rev. Lett.* **2021**, *126* (12), 123002.
5. Zhu, G.-Z, et. al. *Nat. Chem.* **2022**, *14* (9), 995–999.
6. Mitra, D., et. al. *J. Phys. Chem. Lett.* **2022**, *13* (30), 7029–7035.
7. Dickerson, C. E.; *J. Phys. Chem. Lett.* **2021**, *12* (16), 3989–3995.
8. I.S. Lim, H. Stoll, P. Schwerdtfeger, *J. Chem. Phys.* **2006**, *124*, 034107.
9. Ivanov, M. V.; Bangerter, F. H.; Wójcik, P.; Krylov, A. I. *J. Phys. Chem. Lett.* **2019**, *21* , 19447–19457.
10. Ivanov, M. V., et al. *J. Phys. Chem. Lett.* **2020**, *11,* 1297–1304.
11. Ivanov, M. V., et al. *J. Phys. Chem. Lett.* **2020**, *11,* 6670–6676
12. Marian, C. M. *Wiley Interdiscip. Rev. Comput. Mol. Sci.* **2012**, *2*(2), 187–203.

3. Finally, this sentence needs to be corrected:
``The somewhat unexpected atom-like transitions supporting optical cycling and cooling in these small molecules have inspired searches for similar transitions in complex polyatomic molecules with an M-O-R structure,10 where M is an alkaline-earth metal atom ionically bonded to oxygen (O) forming an optical cycling center (OCC) and R is a molecular ligand.33–35''

The authors should directly acknowledge the pioneering work of Isaev andd Berger (ref. 9 in the current draft), who were the first to identify this promising motif for the design of OCCs in polyatomic molecules, on the basis of molecular orbital theory. Their insight was quite remarkable and their proposal remains to be the most commonly utilized in the field.

As the referee correctly points out, we did recognize the work of Isaev and Berger, but did not reference them again when discussing the M-O-R structure. We have added ref. 9 in that sentence along with ref. 10 and a few more references, which now reads:

"**The somewhat unexpected atom-like transitions supporting optical cycling and cooling in these small molecules have inspired searches for similar transitions in complex polyatomic molecules with an M-O-R structure,[9,10,34-40] where M is an alkaline-earth metal atom ionically bonded to oxygen (O) forming an optical cycling center (OCC) and R is a molecular ligand. [35,37-40]**"
